# The effects of equine‐assisted activities on execution function in children aged 7–8 years: A randomized controlled trial

**DOI:** 10.1002/brb3.3148

**Published:** 2023-07-13

**Authors:** XiaoDong Cheng, KeXin Zhen, Yongzhao Fan, Qian Tang, Hao Wu

**Affiliations:** ^1^ School of Kinesiology and Health Capital University of Physical Education and Sports Beijing China; ^2^ Department of Physical Education Beijing Foreign Studies University Beijing China; ^3^ College of Public Education Huainan Union University Huainan China

**Keywords:** equine‐assisted activities, execution function, inhibitory control, working memory, cognitive flexibility, children

## Abstract

**Background:**

: This study examines the effects of equine‐assisted activity (EAA) training on executive functioning (EF) (inhibitory control, working memory, and cognitive flexibility) in children aged 7–8 years.

**Methods:**

: Twenty‐Four healthy children aged 7–8 years with a 1:1 ratio of boys to girls were randomly divided into EAA group (EAAG) or control group (CG). The subjects in EAAG were trained for 12 weeks, and CG participated in normal daily activities. All subjects conducted the Flanker, 1‐Back, and More‐odd shifting tasks at rest and recorded the average reaction times (RTs) and accuracy data of each task.

**Results:**

: After 12 weeks of EAA intervention, EAAG showed a highly significant increase (*p* < .01) in mean RTs and accuracy in the Flanker and More‐odd shifting tasks and a highly significant increase (*p* < .01) in accuracy only in 1‐Back.

**Conclusion:**

: These findings suggest that 12‐week EAA training can be effective in improving EF and promoting cognitive performance in children aged 7–8 years.

## Abstract 2

The combination of Astragalus membranaceus and ligustrazine mitigates cerebral ischemia‐reperfusion injury via regulating NR2B‐ERK/CREB signaling". No graphical summary is written for this article, so our team does not elaborate on it specifically. If it is a magazine that must do so, please contact us in time to make changes.

## 1 INTRODUCTION

Executive function (EF), executive control or cognitive control, is a top–down mental process that requires attention and attentive participation and is considered inappropriate, inadequate, or impossible when automated or operated through instinct or intuition (Burgess & Simons, 2005; Espy, 2004; Miller & Cohen, 2001). Studies have shown that EF includes inhibitory control (IC), working memory (WM), and cognitive flexibility (CF) (Diamond, 2013; Miyake et al., 2000), which play crucial roles in higher level cognitive functions, including reasoning and problem‐solving (Amunts et al., 2021). IC refers to the individual's ability to consciously control, inhibit, or override a superior response, or the ability to ignore irrelevant information or distractions in the environment and focus on relevant information (Miyake et al., 2000). WM is the ability to temporarily retain relevant information and enable an individual to manipulate or further process that information (Baddeley, 2012). CF was the ability to, depending on the task requirements, shift attention or response strategies between mental sets (Miyake et al., 2000). Furthermore, it plays an important role in the performance of children at critical periods of growth and development (McClelland & Cameron, 2019).

Sport is a physical activity with a clear goal, governed by formal rules and concerned with physical movement (Contreras‐Osorio et al., 2021). Previous studies have shown a positive association between physical activity and EF (Contreras‐Osorio et al., 2021). Xu et al. (2022) found that the high‐frequency training group showed significant improvements in WM and CF by setting up high‐frequency, low‐frequency, and control groups (CGs) in a basketball intervention. Similarly, the Ishihara et al. (2017) study showed that tennis game instruction had a positive effect on IC and fitness levels, and that longer coordination training was associated with better WM. In addition, Alesi et al. (2016) suggested that the football group at post‐test showed significantly larger gains than the sedentary group on measures of agility, visuospatial WM, attention, planning, and IC. In general, both team sports and individual sports can be effective in improving EF. Therefore, monitoring EF development during childhood is essential.

But scarce studies have examined the link between equine‐assisted activities (EAAs) and EF performance because few studies to date have attempted to assess EF performance using specific EF assessment tasks. However, studies did imply such a link between EAA and cognitive processes associated with EF. For example, Bass et al. (2009) found improvements in attention skills and distractibility, one of the key skills of EF, when they examined 7‐year‐old children with autism who participated in a therapeutic equine program. Similarly, Kaiser et al. (2006) found that child‐reported levels of inattention, as well as parent‐reported levels of inattention and impulsivity, improved following participation in an equine program for sixth‐ and eighth‐grade boys enrolled in a school‐based special education program. Recent findings show that children diagnosed with psychiatric disorders have improved general intelligence as measured by the Ravens Progressive Matrices after completing an 8‐week equine program (Hession et al., 2014). Such correlational studies provide preliminary support for an association between EAA and EF performances. Unfortunately, most studies have been conducted on children with atypical development, and no studies have been found to correlate with normal children aged 7–8 years. Given that physical activity is associated with the performance of EF, EAA as a physical activity should show a similar association.

In summary, there is a paucity of research on the effects of EAA on EF in normal children. So, the primary hypothesis of this study was a positive effect of 12 weeks of EAA on three sub‐components of EFs (IC, WM, and CF) in normal children aged 7–8 years.

## 2 METHODS AND MATERIALS

### 2.1 Subjects and study design

#### 2.1.1 Subjects

This study was a randomized controlled trial study. The trial starts in early April 2022 and ends in late August 2022. Subjects undergo a 12‐week EAA training intervention at the Maple Leaf International School‐Xi'an equestrian base. The number of experimental subjects was calculated by G*Power software (Version No.3.1.9.7.; Franz Faul University Kiel, Germany) (Faul et al., 2007). The specific parameters were set as follows: *α* = 0.05, power = 0.85, effect size = 0.35, statistical test = repeated measures, number of groups = 2, and number of measurements = 2. With this condition set, there were two groups of 11 people each. However, considering the accuracy of the experiment and the reliability of the data, 13 people per group were finally selected. However, 2 people did not complete the full training intervention in the actual training, and the final number of people who completed the experiment was 24. Yet, unlike other sports, EAA has certain requirements for subjects. Inclusion criteria included: (i) age 7–8 years with normal intelligence and no cognitive disorders; (ii) right‐handed; (iii) in good health, without sports injuries and mental illnesses, and taking no medications; (iv) not enrolled in horseback riding training in the last 6 months; (v) no history of horsehair allergies; (vi) no fear of horses, with boldness, strong will, and high interest in horseback riding. Exclusion criteria included: (i) obesity, BMI ≥ 24; (ii) motor impairment and physical disability; (iii) participation in multiple sports training on Saturdays and Sundays; (iv) unwillingness to cooperate with horseback riding–related movements in the experimental intervention; (v) fear of horses.

A total of 24 children aged 7–8 years were recruited from Maple Leaf International School‐Xi'an in a 1:1 boy and girl ratio and divided into an EAA group (EAAG) and a CG for relevant training interventions. The EAAG continued to participate in 12 weeks of EAA training, training to try to dislodge other external factors, such as colds, fevers, and sports injuries, whereas CG undertook normal daily physical education school and did not participate in other physical activities of higher intensity. The institutional ethical committee of the Capital University of Physical Education and Sports, Beijing, China approved all procedures and protocols (No. 2021A41). It should be noted that all subjects were accompanied by their parents as they learned about the contents of the training intervention and signed an informed consent form. Specific information for all subjects is shown in Table 1.

**TABLE 1:** Basic subject information.

Group	Number	Age	Height (cm)	Weight (kg)	BMI (kg/m^2^)
EAAG	12	7–8	127.2 ± 6.2	27.7 ± 3.6	16.5 ± 1.5
CG	12	7–8	128.6 ± 7.2	27.5 ± 3.5	16.2 ± 1.6

Abbreviations: CG, control group; EAAG, equine‐assisted activity group.

#### 2.1.2 Study design

**FIGURE 1:**
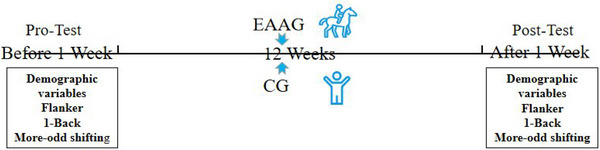
Testing and training timeline.

In this experiment, subjects were numbered and randomized into EAAG and CG by the random function method in Excel 2019, with 12 people in each group, and then a randomized controlled trial design of 2 (group: EAAG, CG)×2 (time: pre‐test, post‐test). A week before the experiment, the pro‐test indicators were tested: demographic variables, Flanker task, 1‐Back, and More‐odd shifting. At the end of the 12‐week intervention, relevant indicators were tested 1 week after the experiment, and the test indicators were consistent with the pro‐test. The testing and training timeline are shown in Figure 1. Importantly, during the monitoring process, the room lights were dimmed, and the room environment was quiet. Furthermore, during the test, subjects were asked to keep their posture stable and remain as still as possible.

### 2.2 Intervention

This experimental protocol was selected from Cook and Frederick's (2016) “Incorporating Play into Hippotherapy A Companion Book to the Brown Pony Series” in improving riders’ cognition, social interaction skills, coordination, and neural control is feasible and effective. With 12 weeks of EAA, 2 training times per week, 45–55 min each, the experimental intervention was conducted from 15:50 to 17:50 p.m. on the first and third of the week. In addition, the experiment was conducted by six MingLiu Horse Club instructors, each responsible for 2–3 children aged 7–8 years. A total of six horses were used in the experiment, each of which was examined by a veterinarian without any health problems and evaluated by a trainer in order to prevent influencing the results of the experiment.

### 2.3 Measures

The IC, WM, and CF were evaluated by Flanker task (Zhan et al., 2020), 1‐Back (Ji & Wang, 2018), and More‐odd shifting (Tian et al., 2021), respectively. The Flanker task, 1‐Back, and More‐odd shifting were designed using E‐Prime 2.0 (Psychology Software Tools Inc.). This task was exhibited on a 15.6‐in. monitor that was 80 cm away from the subjects. The congruent and incongruent reaction times (RTs) and accuracy statistics were performed on the test results at the end of the Flanker task and More‐odd shifting tests. However, the RTs and accuracy were recorded at the end of the 1‐Back test.

#### 2.3.1 Flanker task

In the experimental task, subjects were required to focus on the “+” sign in the middle of the screen to indicate the start of the task. This is followed by a sequence of five letter combinations according to the letters that appear on the screen for 1000 ms, with the gaze point being the position where the middle arrow is located and the stimulus interval being 1 s. This string of letters may appear as follows: congruent conditions, such as “FFFFFF” and “LLLLLL”; incongruent conditions, such as “LLFLL” and “FFLLFF.” The experiment required the subjects to respond to the middle letter as quickly and correctly as possible by pressing the “F” key on the keyboard with the index finger if it was an “F” and the “L” key if it was an “L.” The two conditions were presented equally and randomly, and the formal test consisted of 2 sections, each of which required 60 judgments and 12 practice sessions before the formal test.

#### 2.3.2 1‐Back

During an experimental task, a prompt sign “+” is displayed in the center of the screen to signal the start of that task. In the experimental task, the five letters of the alphabet “B, D, L, Y, and P” were presented as stimuli, and each letter would appear separately in the center of the display, with a stimulus presentation time of 2 s and a stimulus interval of 2 s. Subjects were asked to look at the letters carefully and press the “F” key if the letter presented was the same as the previous one presented, or the “L” key if different. The formal test was divided into 2 segments; each segment had to be completed 24 times and practiced 12 times before the formal test.

#### 2.3.3 More‐odd shifting

Subjects focused their attention on the computer screen, and subjects judged the numbers 1–9 (but not 5), as required, with a numerical duration of 1‐ and a 2‐s interval between two numbers. The task is divided into three parts: the first part, “large/small” judgment: The screen presents a black number, if the number is less than 5, press the “F” key; if the number is greater than 5, press the “L” key. The second part, “odd/even” judgment: The middle of the screen shows green numbers; if it is an odd number, press the “F” key to react; if it is an even number, press the “L” key to react. The third type is “large/small—odd/even” shifting judgment: If the presented numbers are black, “large/small” judgment; if they are green, “odd/even” judgment. Subjects were asked to press the key response as soon as possible while ensuring accuracy. The formal test was divided into six subsections, using the sequence ABCCBA. The A and B sections do not require shifting, 16 times each; the C section requires 32 shifts, which includes 16 shifts. Before the formal start of the exercise, the A and B segments were practiced 8 times each, and the C segment was practiced 16 times.

### 2.4 Statistics and analysis

Descriptive results are reported as means ± standard deviations. The assumption of normality was verified using the Shapiro–Wilk test. The *t*‐test was used to perform the difference test between the EAAG and CG in demographic variables. Statistical analyses of RTS and accuracy associated with the Flanker task, 1‐Back, and More‐odd shifting were performed using a 2 (group: EAAG, CG)×2 (time: pre‐test, post‐test) repeated measures ANOVA. In the case of interaction effects at group × time, simple effects tests were performed. Post hoc analyses using the Bonferroni correction were performed where appropriate. All statistical analyses were performed using the statistical package SPSS 26.0; significant differences are indicated by different letters (^*^ *p* < .05; ^**^ *p* < .01).

## 3 RESULTS

### 3.1 Demographic variables difference examination

Data were investigated and analyzed on individuals in the EAAG and CG samples, where demographic variables included age, height, weight, and BMI, with the purpose of reducing the effect of experimental results due to demographic differences between the two groups, as shown in Table 2.

**TABLE 2:** Demographic data difference comparison.

	EAAG	CG	*t*	*p*
Years	7.5 ± 0.5	7.5 ± 0.5	–	–
Height (cm)	127.2 ± 6.2	128.6 ± 7.2	−.95	.35
Weight (kg)	27.7 ± 3.6	27.5 ± 3.5	.35	.96
BMI (kg/m^2^)	16.5 ± 1.5	16.2 ± 1.6	.31	.82

*Note*: All data were normally distributed (*p* > .05) with chi‐squared (*p* > .05) before independent samples *t*‐tests were performed. In the test of difference between the two groups, height (cm), 127.6 ± 6.2 in EAAG and 128.6 ± 7.2 in CG, *t* = −.95, *p* = .35 > .05; weight (kg), 27.7 ± 3.6 in EAAG and 27.5 ± 3.5 in CG, *t* = .35, *p* = .96 > .05; BMI (kg/m^2^), 16.5 ± 1.5 in EAAG and 16.2 ± 1.6 in CG, *t* = .31, *p* = .82 > .05. The data results showed no differences in demographic variables between EAAG and CG pre‐test.

Abbreviations: CG, control group; EAAG, equine‐assisted activity group.

### 3.2 IC

In this study, the Flanker task data before and after the experimental intervention were statistically analyzed using a 2 (group: EAAG, CG)×2 (time: pre‐test, post‐test) repeated measures ANOVA to analyze the changes of EAA on IC in children aged 7–8 years (see Table 3).

**TABLE 3:** Statistical results of Flanker test variance before and after experimental intervention.

	*df*	*F*	*p*	*η* ^2^partial
Congruent task accuracy (%)				
Time	1	12.83	.001^**^	.25
Time × group	1	9.81	.004^**^	.18
Error	46			
Group	1	.68	.45	.01
Error	46			
Incongruent task accuracy (%)				
Time	1	5.55	.03^*^	.18
Time × group	1	3.42	.07	.09
Error	46			
Group	1	.51	.48	.10
Error	46			
Congruent task RTs (ms)				
Time	1	10.98	.001^**^	.21
Time × group	1	15.68	.000^**^	.32
Error	46			
Group	1	.88	.46	.19
Error	46			
Incongruent task RTs (ms)				
Time	1	42.67	.000^**^	.49
Time × group	1	22.21	.000^**^	.32
Error	46			
Group	1	.87	.41	.02
Error	46			

Abbreviation: RTs, reaction time.

^*^ *p* < .05.

^**^ *p* < .01.

In congruent task accuracy (%), there was no significant main effect of group, *F*(1,46) = .68, *p* = .45 > .05, *η* ^2^partial = .01, whereas there was a significant main effect of time, *F*(1,46) = 12.83, *p* = .001 < .01, *η* ^2^partial = .25, and there was also a significant interaction effect of group × time, *F*(1,46) = 9.81, *p* = .004 < .01, *η* ^2^partial = .18 (see Table 3). A simple effects analysis showed that in the EAAG, there was a highly significant difference (*p* < .01) pre‐and‐post test. In addition, in the EAAG, the congruent task accuracies before and after the experiment were 0.84 ± 0.07 and 0.88 ± 0.06, respectively, whereas in the CG, the congruent task accuracies before and after the experiment were 0.85 ± 0.06 and 0.87 ± 0.05, respectively (see Table 4).

**TABLE 4:** Comparison of Flanker data differences between the two groups.

Test name	Group	Pre‐test	Post‐test
Congruent task accuracy (%)	EAAG	0.84 ± 0.07	0.88 ± 0.06
	CG	0.85 ± 0.06	0.87 ± 0.05
Incongruent task accuracy (%)	EAAG	0.84 ± 0.06	0.87 ± 0.06
	CG	0.85 ± 0.07	0.86 ± 0.06
Congruent task RT (ms)	EAAG	725.27 ± 105.34	682.42 ± 72.55
	CG	718.63 ± 89.45	705.23 ± 71.67
Incongruent task RT (ms)	EAAG	762.17 ± 84.28	689.86 ± 71.39
	CG	752.29 ± 89.68	746.56 ± 91.82

Abbreviations: EAAG, equine‐assisted activity group; CG, control group; RTs, reaction times.

In incongruent task accuracy (%), there was no significant main effect of group, *F*(1,46) = .51, *p* = .48 > .05, *η* ^2^partial = .10, whereas there was a significant main effect of time, *F*(1,46) = 5.55, *p* = .03 < .05, *η* ^2^partial = .18, as shown by post hoc test in the EAAG, there was a highly significant difference (*p* < .01) pre‐and‐post test. However, there was no significant interaction effect for group × time, *F*(1,46) = 3.42, *p* = .07 > .05, *η* ^2^partial = .09 (see Table 3). After 12 weeks of intervention, the EAAGs, before and after the experiment incongruent task accuracy, were 0.84 ± 0.06 and 0.87 ± 0.06, respectively, whereas the CGs, before and after the experiment incongruent task accuracy, were 0.85 ± 0.07 and 0.86 ± 0.06, respectively (see Table 4).

In congruent task RTs (ms), there was no significant main effect of group, *F*(1,46) = .88, *p* = .46 > .05, *η* ^2^partial = .19, whereas there was a significant main effect of time, *F*(1,46) = 10.98, *p* = .001 < .01, *η* ^2^partial = .21, and there was also a significant interaction effect of group × time, *F*(1,46) = 15.68, *p* = .000 < .01, *η* ^2^partial = .32 (see Table 3). The simple effect analysis showed that in the EAAG, there was a highly significant difference (*p* < .01) pre‐and‐post test. In the EAAG, the congruent task RTs before and after the experiment were 725.27 ± 105.34 and 682.42 ± 72.55, respectively, whereas in the CG, the congruent task RTs before and after the experiment were 718.63 ± 89.45 and 705.23 ± 71.67, respectively (see Table 4).

In incongruent task RTs (ms), there was no significant main effect of group, *F*(1,46) = .87, *p* = .41 > .05, *η* ^2^partial = .02, whereas there was a significant main effect of time, *F*(1,46) = 42.67, *p* = .000 < .01, *η* ^2^partial = .49, and there was also a significant group × time interaction effect, *F*(1,46) = 22.21, *p* = .000 < .01, *η* ^2^partial = .32 (see Table 3). The simple effect analysis showed that in the EAAG, there was a highly significant difference (*p* < .01) pre‐and‐post test. In the EAAG, the incongruent task RTs before and after the experiment were 762.17 ± 84.28 and 689.86 ± 71.39, respectively, whereas in the CG, the incongruent task RTs before and after the experiment were 752.29 ± 89.68 and 746.56 ± 91.82, respectively (see Table 4).

### 3.3 WM

In this study, the 1‐Back data before and after the experimental intervention were statistically analyzed using a 2 (group: EAAG, CG)×2 (time: pre‐test, post‐test) repeated measures ANOVA to analyze the changes of EAA on WM in children aged 7–8 years (see Table 5).

**TABLE 5:** Statistical results of 1‐Back variance before and after experimental intervention.

	*Df*	*F*	*p*	*η* ^2^partial
Accuracy (%)				
Time	1	1.51	.27	.04
Time × group	1	3.46	.06	.09
Error	46			
Group	1	.41	.45	.11
Error	49			
RTs (ms)				
Time	1	66.78	.000^**^	.64
Time × group	1	61.56	.000^**^	.56
Error	46			
Group	1	.75	.41	.13
Error	46			

Abbreviation: RTs, reaction times.

^*^ *p* < .05.

^**^ *p* < .01.

In RTs (ms), there was no significant main effect of group, *F*(1,46) = .75, *p* = .41 > .05, *η* ^2^partial = .13, whereas there was a significant main effect of time, *F*(1,46) = 66.78, *p* = .000 < .01, *η* ^2^partial = .64, and there was also a significant interaction of group × time, *F*(1,46) = 61.56, *p* = .000 < .01, *η* ^2^partial = .56, (see Table 5). The simple effects analysis showed that in the EAAG, there was a highly significant difference (*p* < .01) pre‐and‐post test. In the EAAG, the 1‐Back RTs before and after the experiment were 974.82 ± 99.92 and 825.51 ± 84.87, respectively, whereas in the CG, the 1‐Back RTs before and after the experiment were 978.18 ± 97.81 and 968.19 ± 82.64, respectively (see Table 6).

**TABLE 6:** Comparison of 1‐Back data differences between the two groups.

Test name	Group	Pre‐test	Post‐test
Accuracy (%)	EAAG	0.83 ± 0.05	0.85 ± 0.06
	CG	0.83 ± 0.05	0.83 ± 0.06
RTs (ms)	EAAG	974.82 ± 99.92	825.51 ± 84.87
	CG	978.18 ± 97.81	968.19 ± 82.64

*Note*: In accuracy (%), there was no significant main effect of group, *F*(1,46) = .41, *p* = .45 > .05, *η* ^2^partial = .11, and no significant main effect of time, *F*(1,46) = 1.51, *p* = .27 > .05, *η* ^2^partial = .04, and no group × time significant interaction effect, *F*(1,46) = 3.46, *p* = .06 > .05, *η* ^2^partial = .09 (see Table 5). In the EAAG, 1‐Back accuracies before and after the experiment were 0.83 ± 0.05 and 0.85 ± 0.06, respectively, whereas in the CG, 1‐Back accuracy before and after the experiment were 0.83 ± 0.05 and 0.83 ± 0.06, respectively (see Table 6).

Abbreviations: CG, control group; EAAG, equine‐assisted activity group; RTs, reaction times.

### 3.4 CF

In this study, the More‐odd shifting data before and after the experimental intervention were statistically analyzed using a 2 (group: EAAG, CG)×2 (time: pre‐test, post‐test) repeated measures ANOVA to analyze the changes of EAA on CF in children aged 7–8 years (see Table 7).

**TABLE 7:** Statistical results of More‐odd shifting variance before and after experimental intervention.

	*df*	*F*	*p*	*η* ^2^partial
Non‐shifting accuracy (%)				
Time	1	21.38	.000^**^	.31
Time × group	1	30.17	.000^**^	.40
Error	46			
Group	1	2.15	.12	.01
Error	46			
Shifting accuracy (%)				
Time	1	42.71	.000^**^	.46
Time × group	1	30.18	.000^**^	.39
Error	46			
Group	1	.68	.40	.02
Error	46			
Non‐shifting RTs (ms)				
Time	1	11.65	.001^**^	.20
Time × group	1	14.23	.000^**^	.24
Error	46			
Group	1	5.35	.04	.11
Error	46			
Shifting RTs (ms)				
Time	1	15.54	.000^**^	.26
Time × group	1	12.19	.001^**^	.21
Error	46			
Group	1	2.54	.11	.05
Error	46			

Abbreviation: RTs, reaction times.

^*^ *p* < .05.

^**^ *p* < .01.

In non‐shifting task accuracy (%), there was no significant main effect of group, *F*(1,46) = 2.15, *p* = .12 > .05, *η* ^2^partial = .01, and a significant main effect of time, *F*(1,46) = 21.38, *p* = .000 < .01, *η* ^2^partial = .31, and there was also a significant group × time interaction effect, *F*(1,46) = 30.17, *p* = .000 < .01, *η* ^2^partial = .40 (see Table 7). The simple effect analysis showed that in the EAAG, there was a highly significant difference (*p* < .01) pre‐and‐post test. In the EAAG, the non‐shifting task accuracies before and after the experiment were 0.73 ± 0.07 and 0.81 ± 0.06, respectively, whereas in the CG, the non‐shifting task accuracies before and after the experiment were 0.73 ± 0.10 and 0.73 ± 0.09, respectively (see Table 8).

**TABLE 8:** Comparison of More‐odd shifting data differences between the two groups.

Test name	Group	Pre‐test	Post‐test
Non‐shifting accuracy (%)	EAAG	0.73 ± 0.07	0.81 ± 0.06
	CG	0.73 ± 0.10	0.73 ± 0.09
Shifting accuracy (%)	EAAG	0.55 ± 0.08	0.62 ± 0.07
	CG	0.56 ± 0.08	0.57 ± 0.08
Non‐shifting RTs (ms)	EAAG	821.61 ± 103.41	738.56 ± 81.64
	CG	844.12 ± 124.53	839.64 ± 119.24
Shifting RTs (ms)	EAAG	998.68 ± 105.23	928.23 ± 84.41
	CG	1004.19 ± 121.45	997.19 ± 115.29

Abbreviations: EAAG, equine‐assisted activity group; CG, control group; RTs, reaction time.

In shifting task accuracy (%), there was no significant main effect of group, *F*(1,46) = .68, *p* = .40 > .05, *η* ^2^partial = .02, whereas there was a significant main effect of time, *F*(1,46) = 42.71, *p* = .000 < .01, *η* ^2^partial = .46, and there was also a significant interaction effect of group × time, *F*(1,46) = 30.18, *p* = .000 < .01, *η* ^2^partial = .39 (see Table 7). The simple effects analysis showed that in the EAAG, there was a highly significant difference (*p* < .01) pre‐and‐post test. In the EAAG, the shifting task accuracy before and after the experiment were 0.55 ± 0.08 and 0.62 ± 0.07, respectively, whereas in the CG, the shifting task accuracy before and after the experiment were 0.56 ± 0.08 and 0.57 ± 0.08, respectively (see Table 8).

In non‐shifting task RTs (ms), there was a significant main effect of group, *F*(1,46) = 5.35, *p* = .04 < .05, *η* ^2^partial = .11, and a significant main effect of time, *F*(1,46) = 11.65, *p* = .001 < .01, *η* ^2^partial = .20, and a similarly significant group × time interaction effect, *F*(1,46) = 14.23, *p* = .000 < .01, *η* ^2^partial = .24 (see Table 7). The simple effect analysis showed that in the EAAG, there was a highly significant difference (*p* < .01) pre‐and‐post test. In the EAAG, in the non‐shifting task RTs before and after the experiment were 821.61 ± 103.41 and 738.56 ± 81.64, respectively, whereas in the CG, in the non‐shifting task RTs before and after the experiment were 844.12 ± 124.53 and 839.64 ± 119.24, respectively (see Table 8).

In shifting task RTs (ms), there a was significant main effect of group, *F*(1,46) = 2.54, *p* = .11 > .05, *η* ^2^partial = .05, but there was a significant main effect of time, *F*(1,46) = 15.54, *p* = .000 < .01, *η* ^2^partial = .26, and there was also a significant interaction effect of group × time in the switch task response time, *F*(1,46) = 12.19, *p* = .001 < .01, *η* ^2^partial = .21 (see Table 7). The simple effects analysis showed that in the EAAG, there was a highly significant difference (*p* < .01) pre‐and‐post test. In the EAAG, the shifting task RTs before and after the experiment were 998.68 ± 105.23 and 928.23 ± 84.41, respectively, whereas in the CG, the shifting task RTs before and after the experiment were 1004.19 ± 121.45 and 997.19 ± 115.29, respectively (see Table 8).

## 4 DISCUSSION

In our study, changes in EF in children aged 7–8 years were explored primarily through a 12‐week EAA intervention. To our great surprise, 12 weeks of EAA were effective in improving three cognitive tests: Flanker task, 1‐Back, and More‐odd shifting. That is, 12 weeks of EAA improved IC, WM, and CF significantly.

In our research, 12 weeks of EAA were effective in reducing RTs and increasing the accuracy of IC, which is consistent with studies by others (Contreras‐Osorio et al., 2021). Previous studies have shown that the rate of IC development varies at different stages. Anderson et al. (1991) found that 6–7 years old is the sensitive period of development; after 7 years old slow growth, after 10 years old tends to level off. In addition, the subjects in this study belonged to this age group. However, exercise intensity is an important factor in improving postexercise cognitive performance (Chang et al., 2012), further manifested by improved IC performance (Browne et al., 2016). For example, in a meta‐analysis, exercise intensity had a significant effect; the results were positive at 64%–76% or 77%–93% of heart rate max for the prescribed exercise (Chang et al., 2012). Although heart rate was not monitored in this study for riding during exercise, the actual exercise heart rate intensity may be consistent with this intensity. It is important to note that the type of exercise also positively affects IC: chronic exercise (Amatriain‐Fernández et al., 2021; Ludyga et al., 2018). Ludyga et al. assigned adolescents aged 12–15 years to an exercise group and a CG. The exercise group performed 20 min of aerobic and coordinated exercise on study days over an 8‐week period and was shown to enhance IC in adolescents (Ludyga et al., 2018). In terms of overall duration, EAA can also be considered chronic exercise. In addition, we found supportive evidence in a study on open‐ and closed‐skill sports (Formenti et al., 2021). It is commonly recognized that closed‐skill motor activities are performed in a relatively stable and predictable environment in which motor actions are repetitive and unrelated to the external environment; open‐skill physical activity is a dynamic and changing environment, the main feature of which is the motor actions that must be constantly adapted to external stimuli (Di Russo et al., 2010). This change in the context of open‐skill movement, in which inappropriate movements must be inhibited, may be associated with greater challenges in motor skills and activation of brain systems involved in EF (particularly the prefrontal cortex) (Lin et al., 2013). This would imply that the cognitive demands in open skills movements (such as EAA), which are characterized by complex motor movements, may contribute to the explanation of the positive effects of exercise on cognitive function (Best, 2010). It is also important to add that EAA can be effective in improving a rider's concentration (Bass et al., 2009; Ward et al., 2013) and, to some extent, aid in the improvement of IC performance.

The results of the study showed an increasing trend in accuracy and a significant decrease in response time in the 1‐Back task. Related studies have shown some improvement in WM and EF in complex sports, such as martial arts (Giordano et al., 2021), gymnastics (Hsieh et al., 2017), basketball (Xu et al., 2022), and soccer (Wen et al., 2021), and EAA also have more complex technical requirements, so the effect is significant. Furthermore, Krejci et al. (2015) reported an intervention in hippotherapy for children with cerebral palsy and showed some improvement in the attention and memory of the subjects. This in some way suggests that equestrian sports are known to improve children's attention and memory consistent with the present study. In fact, studies have shown that elementary school students are still in a stage of continuous brain development and their nervous system shows plasticity in both micro and macro aspects, which quietly emerges when the individual's own abilities cannot meet the requirements imposed by the environment (Lövdén et al., 2010). As in this study, the rider's pre‐and‐post test accuracy scores on the 1‐Back task were above the standard of 0.8, but to a certain extent, they also showed a mismatch between their own ability and the difficulty of the task, so the subjects kept improving their WM processing efficiency to try to reach the matching state. In addition, general intelligence gains undergo a similar process, increasing with WM efficiency, and recent brain research has provided some evidence that this may be the result of changes in neural network activity within the brain following WM training (Nęcka et al., 2021). In addition, there is also some evidence that physical activity may improve white matter integrity in these brain regions (Chaddock‐Heyman et al., 2014), which in part facilitates improvements in WM. A systematic review and meta‐analysis (Contreras‐Osorio et al., 2021) showed that the frequency of the intervention training was effective in improving WM capacity while being consistent in other sports as well (Xu et al., 2022). The frequency of training one to two times per week in this study was consistent with that of previous researchers.

Exercise as a stressor promotes physiological and psychological arousal (Stork et al., 2018) and increases oxygen and blood flow to the brain (Wheeler et al., 2019), optimizing the allocation of cognitive resources and improving the efficiency of cognitive processing (Chang et al., 2017). This study showed that EAAG performed better on a More‐odd shifting after an EAA intervention compared to a CG. The results are consistent with previous studies (Kujach et al., 2020). There is also evidence that studies in both open and closed skills movement show lower switching costs in the experimental group compared to the CG, suggesting that the open skill movement pattern helps motorists be better prepared for upcoming movement, temporal and spatial transitions and can better switch from one task to another (Tsai & Wang, 2015). As already explained, EAA can be considered an open skills movement, its horse movement is more complex, requiring the rider to constantly change the technical requirements to adapt to the horse's movement, plus the trainer will constantly change the password to put forward new requirements, which requires the rider to make a quick response to the technical action information learned in the brain according to the changes in the external environment, to quickly switch and adjust the technical action carried out by themselves, further improving the conversion ability.

Previous studies have shown that horse‐related activities can increase EF, which is consistent with the present study. First, the horse is motivational and promotes full attention and engagement in the learning situation (Gilboa & Helmer, 2020). Second, the sensory stimulation of ground and riding activities can help regulate the level of physiological arousal and also improve attention and engagement in the activity (Borgi et al., 2016). Finally, the immediate feedback the rider receives from the horse allows for physical and mental self‐regulation (So et al., 2017). It provides different reinforcement for the successful performance of a task and reinforces the learning of the “checking” part of the cognitive strategy. Furthermore, this result can be explained that the improvement of EF by physical activity seems to be related to the physiological changes it causes in the brain. Regular physical activity has been linked to positive changes in brain structure and volume, including increases in white matter, parietal gray matter, the hippocampus, and basal ganglia volume (Benedict et al., 2013; Erickson et al., 2009). In addition, physical activity is thought to affect brain neuroplasticity because it increases BDNF synthesis in the hippocampus, which promotes neuronal and synaptic growth and differentiation and protects against neuronal and synaptic transmission (Lista & Sorrentino, 2010). Moreover, studies show that exercise improves blood circulation to the brain and that aggregation in exercise plasma reduces inflammation and improves memory (De Miguel et al., 2021). The influence may be more pronounced in children aged 6–12, when their brains are rapidly developing, especially in the dorsolateral prefrontal cortex, anterior cingulate cortex, parietal cortex, and subcortical structures like the thalamus, caudate nucleus, nucleus accumbent, and cerebellum (Bidzan‐Bluma & Lipowska, 2018). Therefore, we recommend an increase in EAA for children in this age group to achieve better motor cognition.

Although our findings show some significant effects of EAA on the improvement of EF in children aged 7–8 years, some limitations should be acknowledged. First, our EAAG of subjects were mostly active enthusiasts, and there may be uncontrollable effects of preexisting preferences. Second, the age range was chosen at the stage of 7–8 years old, which is a critical period for cognitive development and has a significant effect; if the age range is increased, this result is not universal. Finally, all our studies have produced behavioral results that do not allow for a deeper explanation of brain neural mechanisms, and useful tools are needed to confirm that cognitive engagement in movement is supporting the development of EF in children.

## 5 CONCLUSION

These findings suggest that a 12‐week EAA intervention can be effective in improving EF (IC, WM, and CF) and promoting cognitive performance in children aged 7–8 years, as well as being a worthwhile physical activity program.

## CONFLICT OF INTEREST STATEMENT

The authors declare that the research was conducted in the absence of any commercial or financial relationships that could be construed as a potential conflict of interest.

### PEER REVIEW

The peer review history for this article is available at https://publons.com/publon/10.1002/brb3.3148.

## Data Availability

The data that support the findings of this study are available from the first author upon reasonable request.
